# The Impact of a Comprehensive Risk Prediction Model for Colorectal Cancer on a Population Screening Program

**DOI:** 10.1093/jncics/pkaa062

**Published:** 2020-07-18

**Authors:** Sibel Saya, Jon D Emery, James G Dowty, Jennifer G McIntosh, Ingrid M Winship, Mark A Jenkins

**Affiliations:** p1 Department of General Practice and Centre for Cancer Research, Faculty of Medicine, Dentistry and Health Sciences, University of Melbourne, Melbourne, Australia; p2 Centre for Epidemiology and Biostatistics, Melbourne School of Population and Global Health, University of Melbourne, Melbourne, Australia; p3 Genomic Medicine and Family Cancer Clinic, Royal Melbourne Hospital, Melbourne, Australia; p4 Department of Medicine, Faculty of Medicine, Dentistry and Health Sciences, University of Melbourne, Melbourne, Australia

## Abstract

**Background:**

In many countries, population colorectal cancer (CRC) screening is based on age and family history, though more precise risk prediction could better target screening. We examined the impact of a CRC risk prediction model (incorporating age, sex, lifestyle, genomic, and family history factors) to target screening under several feasible screening scenarios.

**Methods:**

We estimated the model’s predicted CRC risk distribution in the Australian population. Predicted CRC risks were categorized into screening recommendations under 3 proposed scenarios to compare with current recommendations: 1) highly tailored, 2) 3 risk categories, and 3) 4 sex-specific risk categories. Under each scenario, for 35- to 74-year-olds, we calculated the number of CRC screens by immunochemical fecal occult blood testing (iFOBT) and colonoscopy and the proportion of predicted CRCs over 10 years in each screening group.

**Results:**

Currently, 1.1% of 35- to 74-year-olds are recommended screening colonoscopy and 56.2% iFOBT, and 5.7% and 83.2% of CRCs over 10 years were predicted to occur in these groups, respectively. For the scenarios, 1) colonoscopy was recommended to 8.1% and iFOBT to 37.5%, with 36.1% and 50.1% of CRCs in each group; 2) colonoscopy was recommended to 2.4% and iFOBT to 56.0%, with 13.2% and 76.9% of cancers in each group; and 3) colonoscopy was recommended to 5.0% and iFOBT to 54.2%, with 24.5% and 66.5% of cancers in each group.

**Conclusions:**

A highly tailored CRC screening scenario results in many fewer screens but more cancers in those unscreened. Category-based scenarios may provide a good balance between number of screens and cancers detected and are simpler to implement.

Most countries with guidelines for colorectal cancer (CRC) screening recommend fecal occult blood testing (FOBT), with diagnostic colonoscopy for positive tests, to all within an age range (usually 50-75 years) (1). More intensive screening is recommended for those with higher risks because of their family history of CRC, usually screening colonoscopy instead of FOBT (2,3). Colonoscopy is more sensitive and specific but carries greater cost and risk and is therefore reserved for higher risk individuals. The recommended starting age and frequency of colonoscopy varies between countries, but generally intensity increases with strength of family history. Australian guidelines have 3 broad screening categories that incorporate family history: none or minimal, moderate, or strong family history (Figure 1) (4,5). Most people with CRC do not have a family history, so alone it is not a frequent predictor of disease risk.

**Figure 1.**
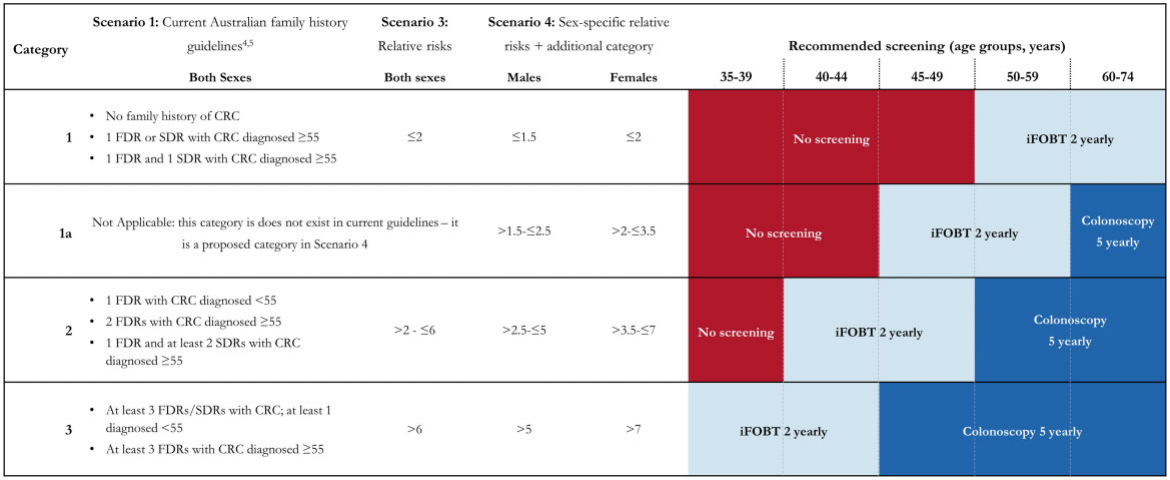
Colorectal cancer (CRC) screening algorithm for current Australian guidelines and 2 proposed scenarios: CRC screening algorithm for scenarios 1 (current Australian guidelines), scenario 3 (using relative risks determined by risk prediction models), and scenario 4 (using sex-specific relative risks determined by risk prediction models, with an additional screening category for those slightly above “average” risk). FDR = first-degree relative; SDR = second-degree relative.

Additional, more common factors influencing future CRC risk provide potential for risk-based screening. These include lifestyle exposures, personal characteristics, rare high-risk genetic variants, and common genomic factors (6–13). Many CRC risk prediction models have been developed, including combinations of these factors; given these exposures’ high prevalence, risk prediction using them is potentially applicable to much more of the population than family history. If these models could be administered to large proportions of a population to estimate personal risk, tailored cancer screening would be possible. This could be more cost-effective than the current model using only family history, because screening could be targeted more efficiently and the burden of false positive FOBT screens or unnecessary screens to those at low risk could be reduced.

Many risk prediction models have been evaluated for their ability to differentiate those who will develop CRC from those who do not by using discrimination measures such as area under the receiver operating characteristic curve (AUROC) (6, 14–16). AUROC has limited clinical relevance and does not reflect a model’s ability to stratify risk within the population (17–20). A better indicator of clinical utility is the model’s ability to identify a small proportion of the population where a large proportion of risk lies. No studies have explicitly explored how existing risk models for CRC stratify risk within the general population.

Other analyses have modeled the impact of genomic and lifestyle models on CRC screening programs, proposing scenarios where each person begins screening when their individual risk of CRC, based on personal risk factors, reaches a predefined threshold (15,16,21–26). These scenarios require substantial tailoring given individuals could potentially begin screening over a wide range of ages, some earlier and some later than the current system. This very tailored program may present not only implementation challenges but also deimplementation challenges; recent studies have shown limited acceptability of beginning screening at a later age despite a known lower risk of cancer (27,28). Additionally, although electronic medical records are increasing in frequency and efficiency, continuous documentation of specific risk information from the time of risk assessment to the commencement of screening may prove difficult with infrastructure upgrades and patient mobility (29). It is possible that simpler screening algorithms that are similar to the current category-based system but incorporate more precise risk prediction may be more practical to implement.

No studies to our knowledge have examined whether a simpler, categorical risk-based model, and one where the latest age for commencing screening is the same as the current system, would result in similar screening efficiency gains to the very tailored programs proposed previously. We explored the impact of a lifestyle and genomic risk prediction model for CRC on screening in the Australian population, proposing more feasible screening algorithms and assessing their impact on the number of people who would be screened and the number of cancers that would occur in those screened groups. This analysis also provides a framework into which newer risk prediction models can be inserted, as they become more predictive and accurate, and provides a basis for the future work that should take place to address the implementation challenges of a risk-stratified CRC screening program.

## Methods

### Study Design

We estimated the distribution of a comprehensive risk models predicted risks in the Australian population (based on family history, lifestyle, and genomic risk factors). We then calculated the number of people and CRC cases recommended to have no screening, immunochemical (iFOBT) screening, and colonoscopic screening under different screening scenarios that are based (in different ways) on predicted risk from the model.

### Risk Prediction Models and Distributions

We calculated the distribution of predicted lifestyle risk using self-reported values for 10 factors from the Colorectal cancer RISk Predictor (CRISP) model (30) for 4747 control participants from the Australasian Colorectal Cancer Family Registry (31) who reported family history of CRC (Supplementary Methods and Supplementary Table 1, available online). Each person’s risk due to family history was based on number, degree of relatedness, and age at diagnosis of relatives with CRC (Supplementary Methods, available online). Each person’s genomic risk was simulated using the theoretical relative risk (RR) distribution in the Caucasian population from 45 CRC-associated single nucleotide polymorphisms (Supplementary Methods and Supplementary Table 2, available online) (24).

To determine the number of people and CRC cases in each screening category, we implemented a mixture of nonparametric and parametric bootstrapping, drawing 500 samples. Each sample of 4000 people was drawn with replacement from the 4747 Australasian Colorectal Cancer Family Registry participants (each having lifestyle and family history risks), then genomic risks were simulated for each by sampling from the genomic risk distribution. Because there is currently no evidence of interactive effects between the 3 types of risk (family history is only weakly associated with these single nucleotide polymorphisms) (15, 32), lifestyle, genomic, and family history relative risks were combined on a log-additive scale. In a supplementary analysis, we examined the genomic and lifestyle models separately (each with family history). Relative risks for CRC were converted to absolute risks using Australian incidences for CRC (33) (Supplementary Methods, available online).

For each bootstrap sample, under each described screening scenario, we calculated the proportion of Australians aged 35-74 years recommended to have no screening, iFOBT screening, or colonoscopic screening. The age limits of 35-74 years were chosen to reflect the earliest and latest ages at which a person may be recommended CRC screening under the current Australian guidelines (5). The proportion of CRC cases expected in each screening category was calculated from absolute risks, with our final estimates being the medians over all bootstrapped samples and 95% confidence intervals being the 2.5th and 97.5th percentiles. Proportions were converted to absolute numbers using the projected Australian population figures for 2020 (34). All analyses were completed in R (35).

### Customizing Screening Based on Risk

We created several implementation scenarios for how CRC risk could be converted to screening recommendations. Scenarios were deliberately designed to reflect previously simulated scenarios (15,21,23) and pragmatic scenarios within limitations of current population screening programs. This resulted in 3 proposals to compare with scenario 1 (Figure 1).

#### Scenario 1:

Current Australian screening guidelines use family history to classify individuals into 3 risk categories (4, 5) developed to incorporate approximate relative risks conferred by constellations of family history (category 1: RR ≤2; category 2: 2 < RR ≤ 6; category 3: RR > 6).

#### Scenario 2:

This scenario provides a highly tailored approach: Screening is based on absolute risk exceeding 2 thresholds. The iFOBT, then colonoscopy, would begin when one’s 10-year risk of CRC exceeds 0.9% (equalling the average 10-year risk of CRC for an Australian aged 50 years, the current starting age for screening) (33) and 4.0% (in current Australian guidelines, this threshold balances cancer risk with risk of complications from colonoscopy) (36), respectively. In 2 sensitivity analyses, these absolute risk thresholds were altered to match the total number of screeners (scenarios 2a) or CRC cases (scenario 2b) as the current guidelines (Supplementary Methods, available online).

#### Scenario 3:

Screening is based on 3 risk categories; the ages when screening is offered mirrors scenario 1, but the screening category is determined by a relative risk threshold calculated using the risk prediction model (Figure 1). The relative risk thresholds were chosen to reflect those used in the current Australian guidelines (category 1: RR ≤ 2; category 2: 2 < RR ≤ 6; category 3: RR > 6).

#### Scenario 4:

Screening is similar to scenario 3, with more precision added as an additional screening category and varying relative risk thresholds for men and women to reflect differing incidence rates by sex (men: category 1: RR ≤ 1.5, category 1a: 1.5 < RR ≤ 2.5, category 2: 2.5 < RR ≤ 5; category 3: RR > 5; women: category 1: RR ≤ 2, category 1a: 2 < RR ≤ 3.5, category 2: 3.5 < RR ≤ 7, category 3: RR > 7).

## Results

### Summary

**Figure 2.**
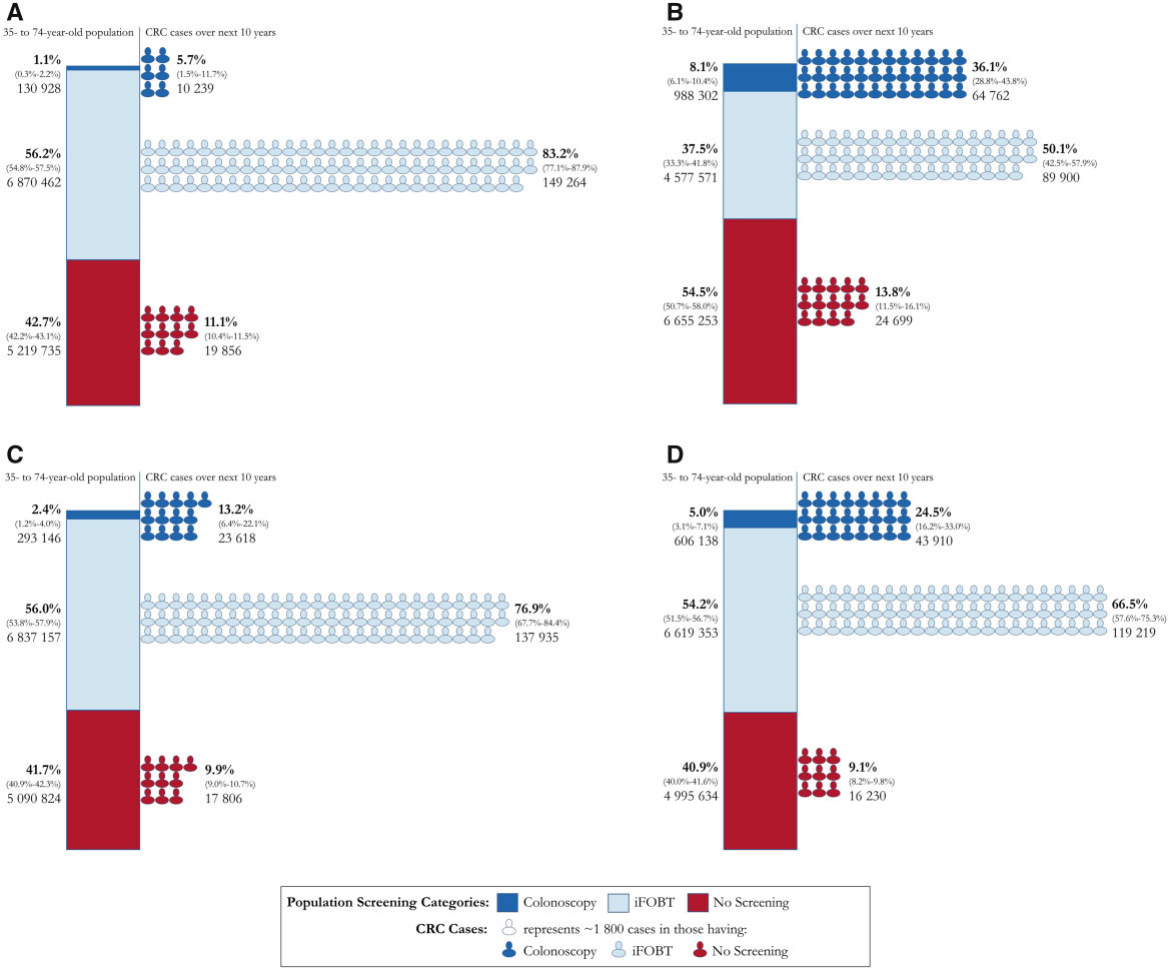
Proportions and number of colorectal cancer (CRC) screens and predicted CRC in each screening group in 35- to 74-year-old Australians. The first column (**bar chart**) in each panel represents the proportion (95% confidence intervals of proportions, absolute number) of 35- to 74-year-old Australians who would not be screened for CRC, be screened with immunochemical fecal occult blood testing (iFOBT), and be screened with colonoscopy under each scenario. The second column (**person icons**) represents the proportion (95% confidence intervals of proportions, absolute number) of predicted CRC in the next 10 years that would occur in each of the screened groups. All scenarios (except scenario 1) use a combined lifestyle and genomic risk prediction model to place individuals in each screening group. **A**) Scenario 1, the current Australian guidelines. **B**) Scenario 2, a program based on absolute risk thresholds for screening using the risk prediction model. **C**) Scenario 3, a category-based program (3 categories not accounting for sex) using the risk prediction model. **D**) Scenario 4, a category-based program (4 categories accounting for sex) using the risk prediction model program. Some percentages do not sum to 100% due to rounding. The 95% confidence intervals for absolute numbers can be found in Supplementary Table 4 (available online).

The proportions and absolute numbers of the Australian population aged 35-74 years who would be recommended screening for CRC via iFOBT and colonoscopy under each scenario, and the respective proportions of CRCs expected in the next 10 years in each of these 3 groups, are shown in Figure 2 (comprehensive model) and Supplementary Figure 1 (available online; genomic and lifestyle model separately).

### Scenario 1: Current Guidelines

Figure 2A shows that 5.7% of CRCs in the next 10 years (10 239 cancers) are expected to occur in 1.1% of 35- to 74-year-olds in Australia (130 928 people) whose age and family history warrant colonoscopy every 5 years, 83.2% of CRCs in the next 10 years (149 264 cancers) are expected to occur in the 56.2% of 35- to 74-year-olds (6 870 462 people) recommended biennial iFOBT screening, and 11.1% of CRCs in the next 10 years (19 856 cancers) are expected in the 42.7% (5 219 735 people) not recommended screening.

### Scenarios Using Risk Prediction Models

Scenario 2 (Figure 2B) adds the risk prediction model. Compared with scenario 1, approximately 8 times more people (approximately 850 000) would be recommended colonoscopy, and approximately 1.4 million more people would not be recommended screening. This tailored scenario would result in a substantial increase in the expected proportion of future CRCs that would occur in the 8.1% recommended the more sensitive colonoscopic screening (36.1% of cancers in next 10 years, 64 762 CRC cases) but also an increase in the proportion occurring in those not screened (13.8%, 24 699 CRC cases would occur in the 54.5% not screened). The remaining 37.5% would be recommended screening with iFOBT, and 50.1% of future CRCs would occur in that group.

Supplementary Table 3 (available online) shows that despite screening the same number of people as in scenario 1, under scenario 2a, more cancers were expected to occur in those recommended colonoscopy compared with scenario 1 (8.0% vs 5.7% of cancers during the next 10 years, translating to 14 296 vs 10 239 CRC cases) and fewer expected cancers in those not being screened (9.0% vs 11.1%, translating to 16 069 vs 19 856 CRC cases). Under scenario 2b, approximately one-half as many people needed to be screened to detect the same number of cancers in the colonoscopy group (70 163 vs 130 928) and fewer in the iFOBT group (6 281 328 vs 6 870 462) compared with scenario 1.

Figure 2, C and D shows scenarios 3 and 4. Under the 2 scenarios based on broader risk categories, the proportion of the population recommended colonoscopy screening (scenario 3: 2.4%; scenario 4: 5.0%) was less than for scenario 2 but more than for scenario 1. Slightly fewer people would be recommended to have iFOBT screening compared with scenario 1 (scenario 3: 56.0%, scenario 4: 54.2%, scenario 1: 56.2%) and fewer cancers would occur in this group (scenario 3: 76.9%, scenario 4: 66.5%, scenario 1: 83.2%). However, slightly fewer cancers would also occur in the nonscreened group for both scenarios 3 and 4 compared with scenario 1 (scenario 3: 9.9%, scenario 4: 9.1%, scenario 1: 11.1%). Therefore, more cancers would be expected to occur in the highest risk colonoscopy group (scenario 3: 13.2%, scenario 4: 24.5%, scenario 1: 5.7%).

## Discussion

This study shows the potential impact on a population screening program of 2 CRC risk prediction models when implemented under different screening scenarios. We show that adding lifestyle and genomic risk to family history and age using simple screening algorithms would identify a larger number of people for screening who are expected to develop CRC. The balance of complexity of the risk stratification process and screening algorithm, number of screens performed, and number of cancers detected warrants consideration. Although this study focuses on impact in the Australian context, these principles can be applied to other populations, particularly countries with population CRC screening programs, reserving the more invasive colonoscopy for those at increased risk because of family history.

A substantial strength of our analysis is that we have incorporated not only the distribution of each individual risk factor (eg, the proportion of the population who eat red meat more than once per day) but also the complex interdependencies between these risk factors, including family history of CRC (eg, the proportion of the population who eat red meat more than once per day and take aspirin and have a family history of CRC). This is unlike other studies that have modeled population CRC risks based on lifestyle exposures (22).

Previous analyses of cancer risk prediction models’ clinical utility have considered scenario 2, where screening would be offered to individuals on reaching an absolute risk threshold (17,21,37). Change from current practice, where most receive the same screening at the same age, to this personalized model could present implementation and deimplementation barriers (29). A lesser shift, potentially more implementable, comprises personalized models that use risk categories (scenarios 3 and 4) not hitherto assessed. We quantify some of the “trade-offs” of a simpler, categorical scenario instead of a highly personalized one.

We demonstrated that a personally tailored model (scenario 2) would substantially reduce the number of total screens (approximately 1.4 million fewer, a 22% decrease) but increase the number of cancers expected to occur in those unscreened (approximately 5000 more cancers over 10 years, a 24% increase). A similar analysis suggested this limitation of risk models to guide cancer screening (21), with reduced screening for those with a low, but nonzero, risk. This is not surprising considering the distribution of cancer risk within the population (38). Although everyone in the sizable portion of the population at the bottom of the distribution has a low risk, which warrants delaying screening to an older age, the sheer volume of people in this category means additional cancers will go unscreened. Following research showing that 85% of women would increase breast screening based on a higher personal genomic risk but fewer (59%) would decrease their screening if found to be low risk (27), this approach may not be acceptable to the general population.

With scenarios 2a and 2b, we directly evaluated the value added by the risk prediction model. These scenarios were like scenario 2 but absolute risk thresholds were set to compare directly to the baseline scenario. Scenario 2a shows that with current screening numbers, a greater proportion of cancers will occur in those screening. Scenario 2b shows that to detect the same number of cancers currently found, fewer people need be screened. This highlights the superiority of the risk prediction model compared with only age and family history and the sensitivity of the impact of a cancer-screening program to risk threshold cutoffs, underlining the importance of modeling screening scenarios.

A better scenario may retain broad risk categories for screening, determining screening category using more accurate risk prediction models than family history alone (scenarios 3 and 4). Everyone older than age 50 would be recommended some screening, in line with current guidelines, potentially decreasing deimplementation challenges of reduction of screening. Although scenarios 3 and 4 resulted in slightly more screening overall (as would any scenario that aims to avoid deimplementation issues), there were more cancers detected in those screened, particularly those with colonoscopy.

This analysis also allowed direct comparison of the lifestyle model, genomic model, and combined risk prediction model. Although the combined risk prediction model always resulted in more cancers occurring in screened groups than each model alone, its implementation is likely to be more laborious, requiring both genomic analysis and collection of lifestyle risk factors. Neither the genomic nor the lifestyle model alone surpassed the other (numbers of cancers predicted to be detected by screening); other logistical aspects of implementing each model warrant consideration if choosing to implement only one. Several studies have already examined the feasibility of administering personalized cancer risk information to the general public within primary care or family practice (39–41) and within centralized cancer-screening programs (42), suggesting they may be feasible. Nonetheless, additional implementation research is required to understand how risk-stratified screening, using genomic and/or lifestyle models, can be embedded in routine care. Other analyses in colorectal (22) and breast cancer (43) demonstrated that the potential for the greatest risk reductions is in those at the highest genomic risk; a combined risk model, with targeted behavioral and screening interventions to those with highest genomic risk, may be optimal.

This study is modeled and therefore based on expected numbers of future cases. This relies on the important assumption that the risk prediction models are well calibrated. This assumption has been found true for the CRISP model in the Australian population (32) but, to the best of our knowledge, not for genomic CRC risk prediction models. Calibration is less studied (15,16,18,20), but several genomic breast cancer risk prediction models are well calibrated (44,45). The methods to develop these are comparable with our genomic model, inferring that our model could show a similar level of calibration. Each of the lifestyle and genomic models has been separately internally and externally validated but not the combined model. Despite these limitations, we provide practical findings for potential clinical impact of this model. Calibration studies of genomic models and validation studies of comprehensive models should be a priority in the future.

The CRISP and genomic models, like many risk prediction models, have been developed primarily from data collected from those of Caucasian ethnicity (6,46). There are important efforts to redress this imbalance in new studies, particularly in the development of genomic tests (47,48). When these more generalizable models are developed, they could be incorporated into future analyses using similar methods to ours.

These models and scenarios in this analysis assume 100% uptake of the risk assessment and recommended screening not consistent with current uptake (1,49). An important aspect of clinical utility is to determine the efficacy of a genomic test “to bring about the intended purpose … when used under the most favourable circumstances” (50) to lead into effectiveness studies examining improvement in outcomes in real-world scenarios. Future studies modeling varying uptake rates of the risk assessment, iFOBT, and colonoscopy screening tests would be useful, ideally based on data from effectiveness studies.

This analysis provides a framework into which more sophisticated risk prediction models can be incorporated. However, it also underlines that there are still noteworthy challenges to be overcome before risk-stratified screening is implementable. Risk prediction models are constantly becoming better calibrated, are more accurate in their risk prediction, their costs are reduced, and are more applicable to all ethnicities, which will go some way to ensuring a cost-effective and equitable future risk-stratified screening program.

Other questions regarding when risk assessment would take place warrant answering. One option could be a central system built within existing population screening programs to facilitate a seamless translation of risk assessment to screening recommendations. Another would see general practitioners performing the risk assessment, creating the opportunity to discuss and then manage modifiable risk factors. The latter approach may also result in greater uptake of the risk assessment and resulting screening recommendations, as demonstrated by studies involving general practitioner endorsement of iFOBT (51,52). Potential barriers to this option include upskilling the current workforce and integration into current general practitioner workflows (53). Future implementation research could determine the approach with the greatest public health impact.

Any risk-stratified screening program would likely need to be dynamic to account for improvements in the risk prediction models, changes in incidence rates (eg, the increasing incidence in younger adults and the impact of screening programs) (54–56), and changes in population structure. It is difficult to predict the effect of these differences on a risk-stratified program, particularly given it is still unclear what is driving the increases in incidence in young people. If these changes are due to differences in environmental exposures, which has been suggested (56), then this will need to be reflected in all parameters in the current model. When this is elucidated, the model we present here could be updated for future analyses.

The different sensitivities of screening modalities warrant examination when modeling the potential gains from a tailored screening program. Colonoscopy, although riskier, has an approximately 95% sensitivity for CRC, where iFOBT is approximately 83% sensitive (57). The proportion of cancers that could be screen detected according to these relative sensitivities would increase from 74.3% in scenario 1 to 77.4% in scenario 2a (the scenario where no more screens are performed). A formal cost-effectiveness analysis—considering the relative sensitivities, specificities, and costs of iFOBT, colonoscopy, and risk assessment consultations—is required to determine if any of these tailored screening scenarios would be cost-effective and could incorporate the potential for genomic risk assessment to include other diseases where prediction models are available. Capacity within the health system for any additional screens required under new risk-stratified programs would also need to be considered.

This clinical utility of any risk prediction model varies, as does AUROC. As risk models improve in their precision, so will their clinical utility, but utility is also affected by the method of implementation. This means that as models are developed, additional to traditional statistics of predictive accuracy, alternative evaluation measures are warranted with exploration of how models might be delivered to the public. This analysis provides only a starting point; an estimate of the clinical utility and potential impact of 2 existing risk prediction models for CRC in screening scenarios that compared with the precise scenarios explored in previous studies may be achievable in the real world. Future analyses using our methods could incorporate many different variables, including updated risk prediction models (which are more applicable to diverse ethnicities and better calibrated), and changes in incidence and demographics, which in turn can feed into cost-effectiveness analyses that incorporate ideal risk thresholds for screening.

## Funding

This work was supported by the Australian Government Research Training Program Scholarship scheme [SS]; the National Health and Medical Research Council Practitioner Fellowship scheme [JDE]; the National Health and Medical Research Council Senior Research Fellow scheme [MAJ]; the National Cancer Institute of the National Institutes of Health [U01CA167551]; and through cooperative agreements with the Australasian Colorectal Cancer Family Registry (the National Cancer Institute of the National Institutes of Health [U01 CA074778 and U01/U24 CA097735]).

## Notes

**Role of the funder:** The funder played no part in the design, analysis, or drafting of this manuscript.

**Conflicts of interest:** Genetype Pty Ltd has provided research support to MAJ, JGM, and JDE, though Genetype Pty Ltd played no part in the design, analysis, or drafting of this manuscript. The authors declare no other competing interests.

**Role of the authors:** Conceptualisation: all authors; data curation: SS and MAJ; formal analysis: SS, JDE and MAJ; investigation: SS and MAJ; methodology: all authors; project administration: SS; resources: MAJ; supervision: JDE, JGM, IMW, JDE and MAJ; validation: JDE and MAJ; visualization: SS and MAJ; writing—original draft: SS; writing—review & editing: all authors.

**Acknowledgments:** The authors would like to acknowledge assistance with collation of data from the Australasian Colorectal Cancer Family Registry and statistical advice from the Melbourne Statistical Consulting Platform, University of Melbourne.

**Data availability statement:** The data that support the findings of this study are available from the corresponding author upon reasonable request.

**Prior presentations:** This article was presented at the following meetings: Cancer in Primary Care Research International Network (Ca-PRI), April 2018, Groningen, The Netherlands; Primary Care Collaborative Cancer Clinical Trials Group (PC4) Conference, May 2018, Sydney, Australia; International Society for Gastrointestinal Hereditary Tumours (InSiGHT), March 2019, Auckland, New Zealand.

## Supplementary Material

pkaa062_Supplementary_Data
