# Supplementary material for: The Impact of a Comprehensive Risk Prediction Model for Colorectal Cancer on a Population Screening Program
Source: JNCI Cancer Spectr. 2020 Jul 18;4(5):pkaa062. doi: 10.1093/jncics/pkaa062 (PMC7583148; doi:10.1093/jncics/pkaa062)
Supplement: pkaa062_Supplementary_Data [file pkaa062_supplementary_data.pdf]

SUPPLEMENTARY METHODS

Lifestyle and family history risk distribution

The CRISP model is a risk prediction model developed using colorectal cancer (CRC) cases and controls of the Colon Cancer Family Registry(1) and includes ten lifestyle risk factors, plus family history of CRC (Supplementary Table 1)(2). The model has a reported AUROC for colorectal cancer of 0.69 and 0.70 for men and women respectively and has been shown to be well calibrated(2).

To determine the distribution of CRISP lifestyle risk in the Australian adult population, we used questionnaire and family history data collected from a subset (4 747 participants) of the Australasian Colorectal Cancer Family Registry. Details on this cohort and their recruitment has been reported elsewhere(1). The subset of participants for this study was restricted to those recruited from population sources and without a personal history of colorectal cancer, and there was also no overlap with the subset used to develop the CRISP model. Using data from this cohort allowed estimation of, not only the frequencies of each category of risk factor (for example, the proportion of the population who eat red meat more than once per day) but cross-tabulations for categories of all risk factors together, including family history of CRC (for example, the proportion of the population who eat red meat more than once per day and take aspirin and have a family history of CRC).

Relative risk conferred from a family history of disease can also be incorporated into the model in a variety of manners as outlined in Zheng et al.(2). In this analysis, risk from family history of CRC was calculated by first calculating the relative risk for constellations of family history according to the formula:

$$RR = e^{\left(0.63*\left(n(FDR \geq 55)+2*n(FDR<55)\right)-0.08\right)} * e^{\left(0.315*\left(n(SDR \geq 55)+2*n(SDR<55)\right)-0.08\right)}$$

where FDR is a first-degree relative with colorectal cancer, SDR is a second-degree relative and "≥55" and "<55" refer to ages of diagnosis. This method of incorporating risk from family history was chosen to balance a simple way to collect this information from patients with the fact that collecting additional detail will result in greater precision of risk estimates. The numbers appearing in this formula were set so that the relative risks for specific family histories were consistent with those reported for a very large US family history study(3) and the mean relative risk over the whole population is 1. Additionally, we assumed that the effect on risk of having a relative diagnosed under age 55 is approximately twice as large as a relative being diagnosed over or at age 55(4); and the effect on risk of having at least one FDR with CRC is approximately twice as large as having at least one SDR with CRC(3). While there is a lot of evidence for the increase in risk for having a FDR with CRC(3-5), there is very little evidence comparing the increased risk conferred by a FDR compared to a SDR. The doubling in risk is consistent with the data provided by Taylor et al.(3). In addition, we assumed that 7% of colorectal cancers in Australia occur in those aged under 55(6), and that 10% of the population has a first-degree-relative with colorectal cancer and therefore 20% has a second-degree-family history(7). This estimate is based on a large cohort study of men and women conducted before widespread CRC screening(7) and the fact that the cumulative risk for CRC in Australia until age 70 is approximately 5.0% for men and women.

This Australasian Colorectal Cancer Family Registry study population was weighted to match the Australian population for age, sex, categories of BMI, smoking status and family history of colorectal cancer(7,8). Frequency of family history was determined under the assumptions stated above. We used the R function rake

from the survey package(9,10) to incorporate cross-tabulations of weighting factors that are not independent of each other (in this case, age-sex-BMI and age-sex-smoking status). This method allowed us to infer the frequency of each combination of CRISP risk factors, including any dependence on family history, in the Australian population, rather than relying on disparate estimates of risk factor frequencies and correlations.

Genomic risk distribution

Genomic risk was based on 45 single nucleotide polymorphisms (SNPs) previously reported to be associated with colorectal cancer(11). The distribution of relative risks was derived from the published population allele frequencies in Caucasian population and their associated per-allele relative risks of colorectal cancer.

In an external validation study, this polygenic risk score had an AUROC for colorectal cancer of 0.63(11). Thus far, to the best of our knowledge, no calibration studies of colorectal cancer polygenic risk scores have been published.

Absolute risk of colorectal cancer

The cumulative risk $CR(t)$ to age t years was calculated as:

$$CR(t) = 1 - \exp\left(-\int_0^t RR\,\lambda(s)ds\right),$$

where $\lambda(s)$ is the relevant population incidence at age s years, based on the age- and sex-specific incidences for Australia(6). Then the probability that an unaffected person who is t years old will develop colorectal cancer within the next 10 years was calculated as:

$$10\,year\ risk = \frac{CR(t+10) - CR(t)}{1 - CR(t)}.$$

Sensitivity analyses

We amended scenario 2 by altering the absolute risk thresholds for iFOBT and colonoscopic screening for ease of comparison to the current program. In scenario 2a, the thresholds are amended to fix total number of people in each screening group to match scenario 1 (current guidelines). This allows direct comparison of the number of cancers in each screening group using the risk prediction model compared with the current scenario. In Scenario 2b, the thresholds are amended to fix the number of cancers in each screening group to match scenario 1 (current guidelines). This allows direct comparison of the numbers needed to screen using the risk prediction model compared with the current scenario.

SUPPLEMENTARY TABLES

Supplementary Table 1: Lifestyle risk factors and associated relative risks (RR) included in the CRISP colorectal cancer risk prediction model

Risk Factor	Categories	Female RR	Male RR
**Body Mass Index**	<25	0.86	0.77
	25-29.9	1.04	1.03
	≥30	1.26	1.25
**Red Meat Intake**	<1/day	0.91	0.96
	≥1/day	1.38	1.18
**Calcium Supplement Usage**	Non-user	1.06	1.00
	≤2.5 years	1.03	1.31
	>2.5 years	0.86	0.62
**Fruit Intake**	<1/day	1.18	1
	≥1/day	0.97	1
**Smoking History**	Never	0.96	0.87
	≤10 pack years	1.08	0.89
	11-20 pack years	1.12	1.00
	21-30 pack years	1.16	0.90
	≥30 pack years	0.95	1.31
**Previous iFOBT**	Never	1.11	1.06
	Ever	0.83	0.86
**Previous Bowel Polyp**	Never	0.98	0.98
	Ever	1.47	1.41
**Previous Colonoscopy**	Never	1.14	1.11
	Ever	0.45	0.55
**Hormone Replacement Therapy Usage**	Never	0.91	N/A
	Oestrogen only	1.26	N/A
	Oestrogen and progesterone	0.93	N/A
	Both oestrogen only and oestrogen and progesterone	1.08	N/A
**Non-Steroidal Anti-Inflammatory Usage**	Never	1	1.08
	≤2 years	1	1.00
	>2 years	1	0.77

Supplementary Table 2: Distribution of number of single nucleotide polymorphisms and associated relative risks. Adapted from Jenkins et al.(11)

Number of Risk Alleles (minimum 0, maximum 90)*	Frequency (%)	Relative Risk
**21**	0.001	0.17
**22**	0.001	0.22
**23**	0.002	0.24
**24**	0.008	0.11
**25**	0.02	0.16
**26**	0.04	0.25
**27**	0.09	0.23
**28**	0.19	0.25
**29**	0.37	0.28
**30**	0.67	0.32
**31**	1.1	0.36
**32**	1.8	0.39
**33**	2.7	0.44
**34**	3.8	0.49
**35**	5.1	0.55
**36**	6.5	0.60
**37**	7.7	0.68
**38**	8.8	0.75
**39**	9.3	0.84
**40**	9.4	0.93
**41**	9.0	1.04
**42**	8.1	1.16
**43**	6.9	1.28
**44**	5.6	1.43
**45**	4.3	1.60
**46**	3.1	1.76
**47**	2.1	1.97
**48**	1.3	2.22
**49**	0.84	2.47
**50**	0.48	2.71
**51**	0.27	3.02
**52**	0.13	3.57
**53**	0.06	4.10
**54**	0.03	4.30
**55**	0.01	4.73
**56**	0.005	5.67
**57**	0.002	9.13
**58**	0.001	5.67
**59**	0.0003	6.67

*Risk allele numbers which have frequencies less than 0.0003% have been omitted given their rarity.

Supplementary Table 3: Estimates and 95% confidence intervals for proportions and number of CRC screens and predicted CRCs in each screening group in 35-74-year-old Australians for scenarios 2a and 2b. Some percentages do not sum to 100% due to rounding.

			35-74-year-old population				Expected colorectal cancers within 10 years			
**Scenario**	**Model**	**Screening Group**	**Estimate (%)**	**95% Confidence Interval (%)**	**Estimate (n)**	**95% Confidence Interval (n)**	**Estimate (%)**	**95% Confidence Interval (%)**	**Estimate (n)**	**95% Confidence Interval (n)**
2a	Combined lifestyle and genomic	No screening	42.8%	(39.4%, 46.1%)	5 225 250	(4 816 223, 5 631 025)	8.0%	(6.6%, 9.4%)	14 296	(11 915, 16 932)
		iFOBT	56.2%	(52.5%, 69.8%)	6 869 137	(6 415 551, 7 304 826)	83.1%	(74.6%, 89.3%)	148 995	(133 785, 160 152)
		Colonoscopy	1.0%	(0.4%, 2.1%)	126 739	(50 260, 253 037)	9.0%	(3.7%, 17.1%)	16 069	(6 669, 30 631)
2b	Combined lifestyle and genomic	No screening	48.0%	(44.3%, 51.6%)	5 869 634	(5 411 916, 6 308 940)	10.3%	(8.5%, 12.0%)	18 437	(15 324, 21 601)
		iFOBT	51.4%	(47.7%, 55.2%)	6 281 328	(5 824 881, 6 745 067)	84.0%	(76.7%, 89.4%)	150 635	(137 509, 160 415)
		Colonoscopy	0.6%	(0.1%, 1.3%)	70 163	(15 261, 159 504)	5.7%	(1.6%, 12.5%)	10 289	(2 892, 22 417)

Supplementary Table 4: 95% confidence intervals for numbers of CRC screens and predicted CRCs in each screening group in 35-74-year-old Australians for each scenario.

Scenario	Model	Screening Group	95% confidence interval for numbers in screening group	95% confidence interval for number of cancers in screening group
1: Current Guidelines	Nil	No screening	(5 155 192, 5 261 455)	(18 646, 20 630)
		iFOBT	(6 700 207, 7 025 194)	(138 353, 157 717)
		Colonoscopy	(35 301, 271 366)	(2 693, 20 939)
2	Combined lifestyle and genomic	No screening	(6 191 897, 7 092 129)	(20 690, 28 891)
		iFOBT	(4 069 945, 5 112 674)	(76 172, 103 790)
		Colonoscopy	(744 206, 1 275 351)	(51 609, 78 559)
	Lifestyle	No screening	(5 993 310, 6 745 370)	(21 819, 28 738)
		iFOBT	(4 439 756, 5 413 856)	(87 259, 112 088)
		Colonoscopy	(700 174, 1 181 809)	(43 165, 66 320)
	Genomic	No screening	(5 963 139, 6 680 243)	(21 690, 28 927)
		iFOBT	(4 490 460, 5 469 107)	(85 317, 111 861)
		Colonoscopy	(707 468, 1 191 144)	(43 543, 68 012)
3	Combined lifestyle and genomic	No screening	(4 997 138, 5 172 643)	(16 199, 19 194)
		iFOBT	(6 571 760, 7 074 061)	(121 455, 151 386)
		Colonoscopy	(145 674, 490 807)	(11 446, 39 560)
	Lifestyle	No screening	(5 111 695, 5 237 936)	(18 343, 20 386)
		iFOBT	(6 720 675, 7 070 267)	(137 241, 158 320)
		Colonoscopy	(36 281, 268 974)	(2 557, 22 100)
	Genomic	No screening	(5 090 996, 5 231 104)	(17 564, 20 122)
		iFOBT	(6 626 405, 7 048 641)	(129 882, 155 215)
		Colonoscopy	(77 914, 371 667)	(6 335, 29 928)
4	Combined lifestyle and genomic	No screening	(4 883 532, 5 089 309)	(14 659, 17 628)
		iFOBT	(6 290 583, 6 932 101)	(103 332, 135 019)
		Colonoscopy	(382 390, 868 257)	(29 123, 59 131)
	Lifestyle	No screening	(5 000 767, 5 168 183)	(16 844, 19 061)
		iFOBT	(6 532 317, 7 032 341)	(123 925, 150 064)
		Colonoscopy	(177 109, 540 552)	(12 260, 36 825)
	Genomic	No screening	(4 988 004, 5 167 469)	(16 280, 19 062)
		iFOBT	(6 349 724, 6 907 919)	(113 577, 141 601)
		Colonoscopy	(293 825, 733 526)	(20 959, 47 331)

SUPPLEMENTARY FIGURE

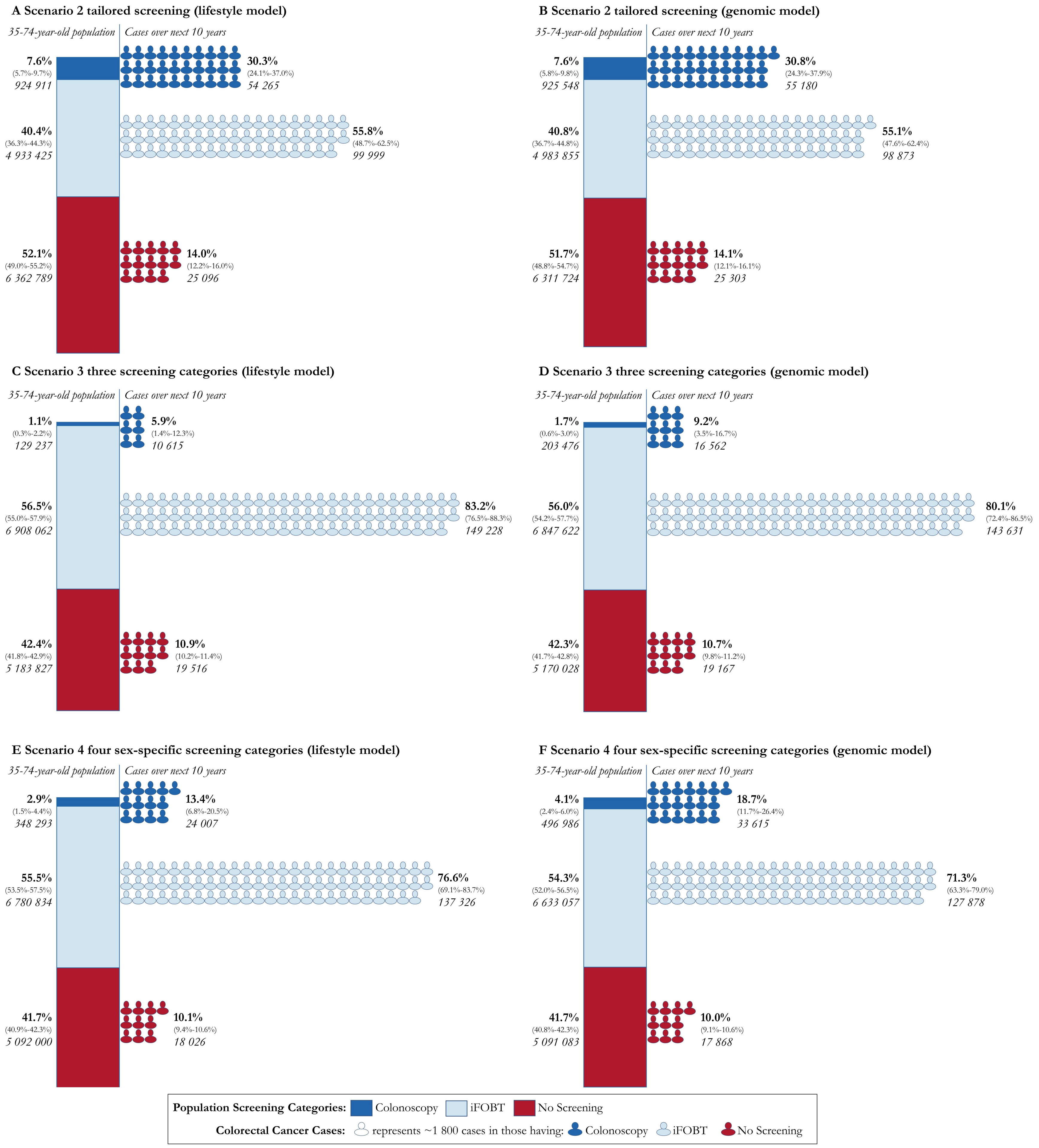

Supplementary Figure 1: **Proportions and number of CRC screens and predicted CRCs in each screening group in 35-74-year-old Australians for the lifestyle and genomic risk predictions models alone:** The first column (bar chart) in each panel represents the proportion (95% confidence intervals of proportions, absolute number) of 35-74-year-old Australians who would not be screened for CRC, be screened with iFOBT, and be screened with colonoscopy under each scenario. The second column (person icons) represents the proportion (95% confidence intervals of proportions, absolute number) of predicted CRCs in the next ten years that would occur in each of the screened groups. Panels A), C), and E) use a lifestyle risk prediction model and panels B), D), and F) use a genomic risk prediction model to place individuals in each screening group. Panels A) and B) scenario 2, a program based on absolute risk thresholds for screening using each risk prediction model; panels C) and D) scenario 3, a category-based program (3 categories not accounting for sex) using each risk prediction model; panels E) and F) scenario 4, a category-based program (4 categories accounting for sex) using each risk prediction model. Some percentages do not sum to 100% due to rounding.

SUPPLEMENTARY REFERENCES

1. Jenkins MA, Win AK, Templeton AS, *et al.* Cohort Profile: The Colon Cancer Family Registry Cohort (CCFRC). Int J Epidemiol 2018;47(2):387-388i.
2. Zheng Y, Hua X, Win AK, *et al.* A new comprehensive colorectal cancer risk prediction model incorporating family history, personal characteristics, and environmental factors. Cancer Epidemiology and Prevention Biomarkers 2020.
3. Taylor DP, Burt RW, Williams MS, *et al.* Population-based family history-specific risks for colorectal cancer: a constellation approach. Gastroenterology 2010;138(3):877-85.
4. Baglietto L, Jenkins MA, Severi G, *et al.* Measures of familial aggregation depend on definition of family history: meta-analysis for colorectal cancer. J Clin Epidemiol 2006;59(2):114-24.
5. Johns LE, Houlston RS. A systematic review and meta-analysis of familial colorectal cancer risk. The American Journal of Gastroenterology 2001;96(10):2992-3003.
6. Australian Institute of Health and Welfare. Australian Cancer Incidence and Mortality (ACIM) books: colorectal cancer. In: AIHW, (ed). Canberra, Australia; 2016.
7. Fuchs CS, Giovannucci EL, Colditz GA, *et al.* A prospective study of family history and the risk of colorectal cancer. New England Journal of Medicine 1994;331(25):1669-1674.
8. Australian Bureau of Statistics. *Australian Health Survey: Updated Results, 2014-2015*. http://www.abs.gov.au/ausstats/subscriber.nsf/log?openagent&4364055001do008_20142015.xls&4364.0.55.001&Data%20Cubes&4B20A1660EBE0012CA257F150009F9F3&0&2014-15&08.12.2015&Latest.
9. R Core Team. R: A language and environment for statistical computing. In: Computing RFfS, (ed). Vienna, Austria; 2016.
10. Lumley T. Analysis of complex survey samples. Journal of Statistical Software 2004;9(1):1-19.
11. Jenkins MA, Makalic E, Dowty JG, *et al.* Quantifying the utility of single nucleotide polymorphisms to guide colorectal cancer screening. Future Oncol 2016;12(4):503-13.
